# The Long-Term Effectiveness of Methadone Maintenance Treatment in Prevention of Hepatitis C Virus Among Illicit Drug Users: A Modeling Study

**DOI:** 10.5812/ircmj.13484

**Published:** 2014-02-07

**Authors:** Mehdi Javanbakht, Alireza Mirahmadizadeh, Atefeh Mashayekhi

**Affiliations:** 1 Health Economics Research Unit, Institute of Applied Health Sciences, University of Aberdeen, Aberdeen, UK; 2 Non-Communicable Diseases Research Center, Endocrinology and Metabolism Research Institute, Tehran University of Medical Sciences, Tehran, IR Iran; 3 HIV/AIDS Research Center, Shiraz University of Medical Sciences, Shiraz, IR Iran; 4 Health Management and Economics Research Center, School of Health Management and Information Sciences, Iran University of Medical Sciences, Tehran, IR Iran

**Keywords:** Hepatitis C Virus, Iran, Methadone Maintenance Treatment, Quality Adjusted Life Years, Illicit Drug Users, Markov Model

## Abstract

**Background:**

Chronic infection with hepatitis C virus (HCV) is increasingly recognized as a major global health problem. Illicit injection drug use is an important risk factor for the rising hepatitis C virus (HCV) prevalence in IR Iran.

**Objectives:**

The objective of this study was to determine the long-term effectiveness (total quality adjusted life years (QALYs) gained) of methadone maintenance treatment (MMT program) in prevention of HCV infection among injecting drug users (IDUs).

**Materials and Methods:**

A number of Markov models were developed to model morbidity and mortality among IDUs. The input data used in modeling were collected by a self-reported method from 259 IDUs before registration and one year after MMT and also from previous studies. One way and probabilistic sensitivity analyses were done to show the effects of uncertainty in parameters on number of life years and QALYs saved. The expected consequences were estimated using a life-time time horizon for the two strategies including implementation and not implementation of the MMT program.

**Results:**

Our model estimated that total number of discounted life years lived per IDU with and without the MMT program would be 5.15 (5.05 - 5.25) and 4.63 (4.42 - 4.81), respectively. The model also estimated that total number of discounted QALYs lived per IDU with and without the MMT program would be 4.11 (3.86 - 4.41) and 2.45 (2.17 - 2.84). Simulation results indicated that all differences in life years and QALYs lived between the two strategies were statistically significant (p < 0.001). Based on our model, total discounted life years and QALYs saved in a cohort of 1000 IDUs were 1790 (1520 - 2090) and 1590 (1090- 2090), respectively.

**Conclusions:**

Considering the high prevalence of illicit injecting drug use in Iran and MMT effectiveness in prevention of HCV infection, it is necessary to develop MMT centers at regional and national levels.

## 1. Background

Hepatitis C virus (HCV) is a leading cause of chronic liver disease, as well as the most common chronic blood borne infection worldwide (1, 2). It has been estimated that 2 to 3% of the global population, which corresponds to about 170 million people are now infected with HCV (3, 4). About 70-80% of infected patients develop chronic infection, which leads to hepatic fibrosis, cirrhosis, hepatocellular carcinoma, and death (5, 6). A higher seroprevalence of HCV infection has been reported among people who are injecting drug users (IDUs) (4, 7, 8). It has been estimated that about 1% of the Iranian general population has anti-hepatitis C virus antibodies (9, 10). The range of HCV infection among Iranian IDUs has been estimated to be 34 % to 88 % (4, 11, 12). High number of illicit injecting drug users in Iran as well as high prevalence of HCV infection among IDUs and sharing injecting equipment constitutes an ongoing threat. Iran has started a number of harm reduction programs for tackling HIV, HBV, and HCV epidemics among IDUs. Methadone maintenance treatment (MMT) is by far the most widely available treatment for addiction to heroin and other opiates. Considerable interest on the role of MMT in the treatment of opioid dependency and its potential to reduce criminal activity and other high-risk behaviors has led to recent studies focusing on the effectiveness evaluation of MMT.

## 2. Objectives

Previous studies have shown that providing MMT helps release IDUs from opioid dependency, facilitating better adherence to ART and improving virological outcomes (13-19). Nonetheless these evaluations were mostly carried out in developed countries and there has been little evidence regarding the effectiveness of MMT in prevention of HCV among IDUs in developing countries. To enable comparisons across different interventions in healthcare, a common measure is required. This measure should ideally encompass the impact of an intervention on a patient's length of life and also the impact on their health-related quality of life. The quality-adjusted life year (QALY) has been developed in order to capture both of these impacts and is widely used in health economics as a summary measure of health care outcome, which can be used by health policy makers for healthcare resource allocation decisions. Since there are a few studies regarding assessment of MMT program effectiveness in Iran, we designed the current study to estimate the long-term effectiveness of MMT program in prevention of HCV infection among IDUs.

## 3. Methods and Material

### 3.1. Study Design

This was a modeling study to estimate the long-term effectiveness of MMT program in prevention of HCV infection. A number of Markov models were developed to model morbidity and mortality in a cohort of 1000 IDUs. To estimate the number of new infections in each cycle mathematical modeling was used. Our methods for measuring the incidence of HCV infection with/without the MMT program are described in details elsewhere (20). In brief, we used a mathematical model which was designed by Rehle and colleagues (21) that determines the changes in drug users' possible high risk behaviors and shows the probability of obtaining infection or transmitting infection. The input data used for our mathematical model were collected by a self-reported method from 259 IDUs in 7 governmental MMT centers of Shiraz, south of Iran. Before enrolment, the subjects received detailed written and verbal information regarding the aims and protocols of the study and signed informed consents. The study was approved by the Ethics Committee of the Shiraz University of Medical Sciences. The model results showed that cumulative incidence of HCV per 100 IDUs due to sharing injection and unsafe sexual acts after MMT program were 13.84 (95 % CI: 6.17 - 21.51), 0.0003 (0.0001 - 0.0005) while before the program these values were 36.48 (25.84 - 47.11) and 0.0004 (0.0002 - 0.0006), respectively. Now we used these estimated parameters along with other external parameters to estimate total life years and quality adjusted life years (QALYs) saved as long-term effectiveness of MMT program in prevention of HCV infection.

### 3.2. Estimation of Total Life Years and QALYs Saved

**Figure 1.**
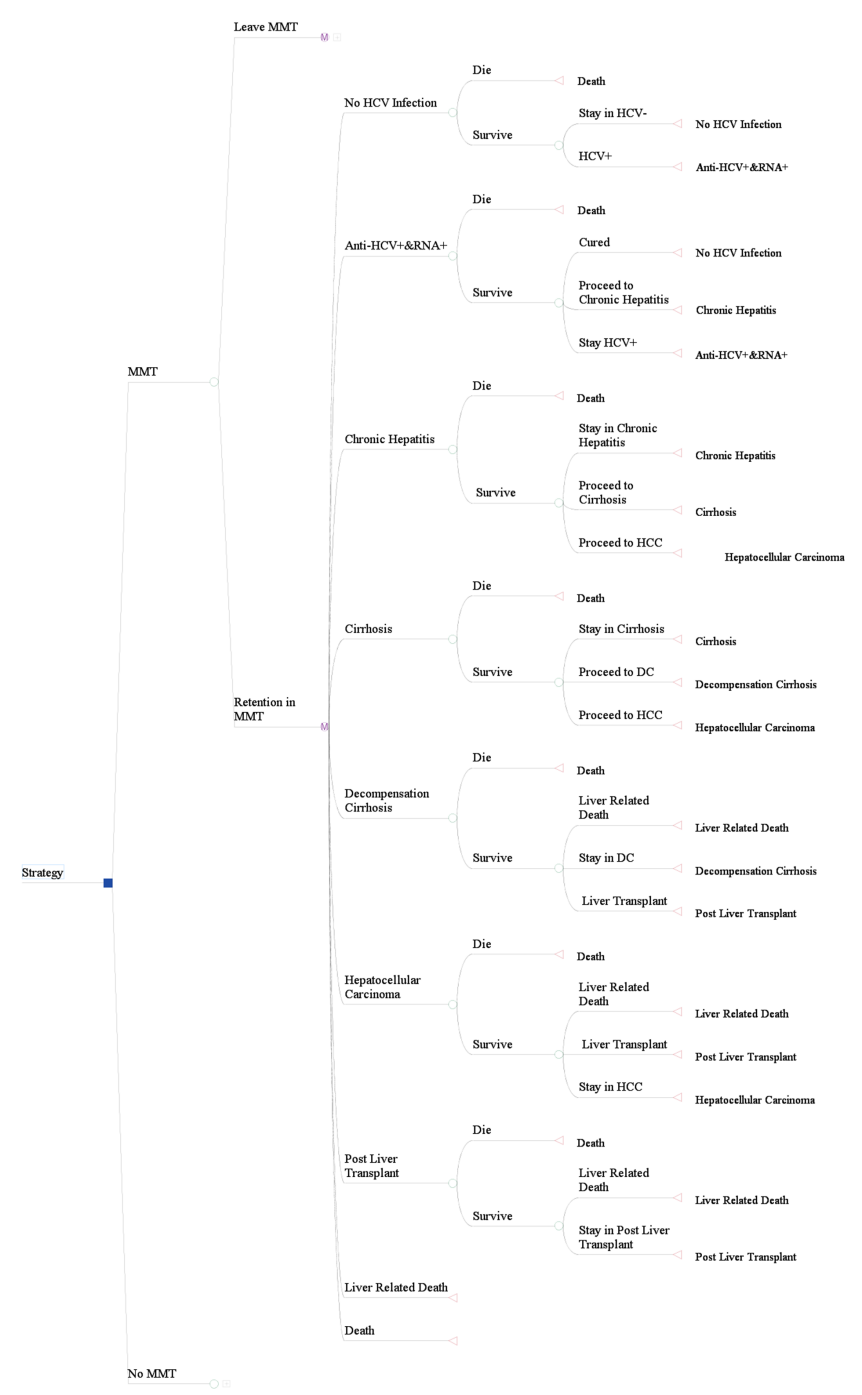
Markov Model Abbreviations: DC: decompensation cirrhosis; HCC: hepatocellular carcinoma. Note: no MMT branch is the same as MMT but with different input parameters.

We constructed our Markov model after infection by HCV with nine health states: HCV-, anti-HCV+ and RNA+, chronic hepatitis, cirrhosis, decompensation cirrhosis, hepatocellular carcinoma, post liver transplant, liver related death and death from background mortality (Figure 1). The expected consequences were estimated using a life-time time horizon (modeling within IDUs life expectancy) for two strategies including implementation and no implementation of the MMT program. After each cycle based on estimation of new cases of HCV infection our model parameters were recalibrated. Linear interpolation was used to estimate interval probabilities where the studies reported a cumulative event rate. Estimated future life years and QALYs were discounted using a 3% annual discount rate. We assumed that transitions between health states occurred annually and their probabilities were derived from previous studies. Annual transition probabilities for each cycle have been calculated using the p = 1- e-rt formula; where p is probability, e is base of natural logarithm, r is event rate and t is time period (22). We simulated a cohort of IDUs with mean age of 33 years (mean age of our sample) through the model until all members of the cohort died. Each IDU in the model may exit through cessation or death, with or without becoming HCV-infected. If infected, the model tracks the development of HCV-related liver disease in the cohort based on transition probabilities. We adjusted the life table of Iran (23) by standard mortality ratio (SMR) for IDUs and their sex and age-specific probability of death were applied to estimate the number of life years and QALYs saved. We also assumed a 55% (50 – 60%) rate of retention in MMT after the first admission. Furthermore, we took in to account the extra rate of mortality and disease progression among IDUs who are co-infected with HCV and HIV. Values of all external parameters and model inputs are shown in Table 1. 

**Table 1.** Input Parameters Used in the Model

Parameters	Min	Base-Case Value	Max	Source
**HCV prevalence among Iranian IDUs (%)**	34	48	68	(4, 11)
**HCV/HIV co-infection prevalence among Iranian IDUs (%)**	8.7	24	35.5	(12, 24)
**Rate of HCV transmission via shared injection (%)**	0.84	4	10	(25-27)
**Number of injections per week without MMT (95% CI)**	19.44	21.28	23.13	(20)
**Number of sharing injections per week without MMT (95% CI)**	2.43	3.1	3.76	(20)
**Number of shared person in each party without MMT (95% CI)**	1.7	2.04	2.38	(20)
**Number of injections per week with MMT (95% CI)**	6.81	7.74	8.67	(20)
**Number of sharing injection per week with MMT (95% CI)**	0.28	0.4	0.52	(20)
**Number of shared person in each party with MMT (95% CI)**	0.27	0.37	0.46	(20)
**Number of unsafe heterosexual contact per month without MMT (95% CI)**	0.56	0.75	0.94	(20)
**Number of unsafe homosexual contact per month without MMT (95% CI)**	0.1	0.2	0.31	(20)
**Number of unsafe heterosexual contact per month with MMT (95% CI)**	.34	0.51	0.69	(20)
**Number of unsafe homosexual contact per month with MMT (95% CI)**	0.02	0.06	0.11	(20)
**Rate of HCV transmission per sexual act**	7 × 10^-8^	5 × 10^-7^	1 × 10^-6^	(28, 29)
**Condom efficacy**	0.35	0.9	0.95	(30, 31)
**Transition Probabilities **	-	-	-	-
Transition from Chronic Hepatitis to Cirrhosis	0.087	0.12	0.16	(2, 32)
Transition from Chronic Hepatitis to HCC^a^	0.0001	0.001	0.02	(32)
Transition from Cirrhosis to Decompensation Cirrhosis	0.032	0.065	0.092	(32, 33)
Transition from Cirrhosis to HCC^a^	0.024	0.033	0.046	(32, 33)
Transition from Decompensation Cirrhosis to Post Liver transplant	0.017	0.033	0.049	(32-34)
Transition from HCC^a^ to Post Liver transplant	0.05	0.1	0.18	(32)
Transition from Decompensation Cirrhosis to Liver Related Death	0.137	0.186	0.25	(32-35)
Transition from HCC^a^ to Liver Related Death	0.32	0.35	0.68	(32-34)
Transition from Post Liver transplant to Liver Related Death (First year)	0.127	0.146	0.21	(32-35)
Transition from Post Liver transplant to Liver Related Death (After first year)	0.035	0.044	0.053	(32-35)
Standardized mortality ratio (SMR) among IDUs (including HIV/AIDS and drug overdose related death)	9.95	14.47	19	(27, 36)
RR^a^ of mortality among IDUs in MMT compared to those who weren’t part of the program (%)	25	32	38	(18, 19, 27)
Retention Rate in MMT (%)	50	55	60	(18, 19, 27)
RR^a^ of progression to chronic hepatitis in co-infected IDUs compared to IDUs mono-infected with HCV	-	1.4	-	(37)
RR^a^ of progression to cirrhosis in co-infected IDUs compared to IDUs mono-infected with HCV	1.05	2.74	7.15	(38)
RR^a^ of progression to DC^a^ in co-infected IDUs compared to IDUs mono-infected with HCV	2.86	6.14	13.2	(39)
CHR^a^ of liver related death in co-infected IDUs compared to IDUs mono-infected with HCV	1.98	7.15	25.8	(40)
**Utility^b^**	-	-	-	-
IDU and asymptomatic HCV + in MMT	0.60	0.72	0.83	(41)
IDU and asymptomatic HCV + not in MMT	0.60	0.65	0.70	(41)
Chronic Hepatitis	0.47	0.54	0.61	(42)
Cirrhosis	0.40	0.46	0.52	(42)
Decompensation Cirrhosis	0.33	0.40	0.45	(42)
HCC^a^	0.31	0.37	0.43	(42)
Post Liver transplant (First year)	0.29	0.33	0.37	(43-45)
Post Liver transplant (After first year)	0.39	0.49	0.58	(43-45)

^a^ Abbreviations: CHR: cause-specific hazard ratio; RR: relative risk; DC: decompensated cirrhosis, HCC: hepatocellular carcinoma.

^b^ Utility score was adjusted based on the Iranian IDUs quality of life.

### 3.3. Sensitivity Analysis

Model robustness was tested in a probabilistic sensitivity analysis (PSA) to determine the effect of all parameters uncertainties simultaneously within the model using Monte Carlo simulation, with a generation of 1000 trials. In each trial, values for model parameters were drawn from pre-specified probability distributions. We assigned beta-distributions for all utilities and most of the transition probabilities, though some transition probabilities were modeled using a triangular distribution. We followed these steps to define distribution types and their parameters in the sensitivity analysis. For model parameters, which were extracted from our primary data we used the Easyfit software to determine the most appropriate distribution and its parameters. For other parameter, which was extracted from the literature based on the parameters nature and its proper span, we assigned appropriate distributions. For example for transition probability and health utilities which were between 0-1, we assigned beta distribution and their parameters including Alpha and Beta where estimated using the following formulas:

Alpha = ((µ^2) × (1- µ)/( σ ^2))

Beta = (µ × (1-. µ)/( σ ^2))-(( µ ^2) × (1- µ)/( σ ^2))

For parameters where only the mean and minimum and maximum were available, we assigned a triangular distribution. We also conducted a deterministic one-way sensitivity analysis in which each parameter was changed in a sequence to the upper and lower limits at a defined range while the other variables were held constant. For statistical analysis, after Monte Carlo simulation within a probabilistic sensitivity analysis the yielded numbers from Monte Carlo trials were checked for normality via Kolmogorov-Simonov test and then Wilcoxon sign rank test was used to compare the means. All models and simulation constructed in Treeage software.

## 4. Results

Of the 259 participants, 98.4% (255) were men and the mean age ± SD was 33.1 ± 7.58 years. Among all studied IDUs 138 individuals had HCV certified clinical tests, which showed that HCV prevalence was 50 %. Our results related to the number life years and QALYs lived per IDU are summarized in Table 2. Our model estimated that the total numbers of undiscounted life years lived per IDU with and without the MMT program were 5.96 (5.84 - 6.05) and 5.26 (5.02 - 5.49), respectively. After discounting, total life years lived per IDU with and without the MMT program were estimated to be 5.15 (5.05 - 5.25) and 4.63 (4.42 - 4.81), respectively. The model also estimated that the total number of undiscounted QALYs lived per IDU with and without the MMT program were 4.89 (4.54 - 5.32) and 2.77 (2.44 - 3.19), respectively. However, when the QALYs lived were discounted, we found that total QALYs lived per IDU with and without the MMT program were 4.11 (3.86 - 4.41) and 2.45 (2.17 - 2.84), respectively. Simulation results indicated that all differences in life years and QALYs lived between the two strategies were statistically significant (P < 0.001).

Based on our model total undiscounted and discounted life years saved in a cohort of 1000 IDUs would be 700 (350 - 1030) and 520 (240 - 730), respectively. In addition the accordant total estimated undiscounted and discounted QALYs were estimated to be 2120 (1350 - 2880) and 1660 (1270- 2240), respectively (Table 3). 

**Table 2.** Total Life Years and QALYs lived per IDU

Outcome Measure	Without MMT			With MMT			P Value
	Mean	95% CI		Mean	95% CI		
		Low	High		Low	High	
**Life years lived per IDU**							
**Not discounted**	5.26	5.02	5.49	5.96	5.84	6.05	< 0.001
**Discounted**	4.63	4.42	4.81	5.15	5.05	5.25	< 0.001
**QALYs lived per IDU**							
**Not discounted**	2.77	2.44	3.19	4.89	4.54	5.32	< 0.001
**Discounted**	2.45	2.17	2.84	4.11	3.86	4.41	< 0.001

**Table 3.** Total Estimated Life Years and QALYs Saved

Outcome Measure	Mean	Range	
		Low	High
**Total life Years saved per 1000 IDU**			
**Not discounted**	700	350	1030
**Discounted**	520	240	730
**Total QALYs saved per 1000 IDU**			
**Not discounted**	2120	1350	2880
**Discounted**	1660	1270	2240

**Figure 2.**
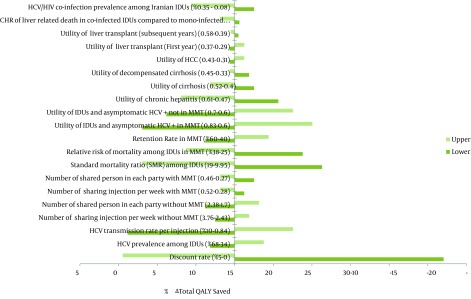
Results of One-Way Sensitivity Analysis (Tornado Diagram)

We also investigated how changes in model parameters would affect the total QALYs saved, as the final outcome using one-way sensitivity. Our results showed that changes in most of the input parameters had a few effects on the total QALYs saved, as the main long-term effectiveness. The total QALYs saved were, especially, highly sensitive to discount rate (adopting 0% and 5% discount rate in the model would change the total QALYs saved by +27% and -14.47%, respectively), HCV transmission rate per injection (changing HCV transmission rate per injection from 4% to 0.84% and 10%, would change the total QALYs saved by -13.84 % and +7.55%, respectively), utility weight among IDUs and asymptomatic HCV + (changing utility of IDUs and asymptomatic HCV + in MMT from 0.72 to 0.6 and 0.83 would change the total QALYs saved by -11.95% and +10.06%, respectively) and SMR among IDUs (changing SMR among IDUs from 14.47 to 9.95 and 19 would alter the total QALYs saved by +11.32 and -11.95, respectively) (Figure 2). 

## 5. Discussion

This study was conducted to examine the long-term effectiveness of the MMT program in prevention of HCV infection among IDUs. Up to now, a number of studies have shown that MMT can reduce high risk injecting and sexual behaviors, which can prevent the incidence of infectious disease among IDUs (13-15, 19, 46-48). It is evident that these kinds of analyses underestimate total health outcome of the MMT program because people can live healthier in subsequent years due to aversion from being infected. Furthermore, due to limited resources in health systems worldwide, demand for economic evaluations of health care programs is steadily increasing. One of the most important issues in this field is to measure effectiveness of interventions by a standard measure of health outcome such as life years and QALY. Besides, measuring impact of interventions in terms of life years and QALY allow us to compare different interventions in health systems. In this study we tried to estimate the total number of life years and QALYs saved as results of low incidence of HCV infection among IDUs who participate in MMT programs. Our model estimated that total life years and QALYs lived are significantly higher among IDUs who receive MMT compared to non-recipients. In agreement with our finding, previous studies have revealed that the MMT program can increase life expectancy of IDUs (19, 27, 49, 50). Our results indicated that IDUs who participated in the MMT program could live, on average 0.7 year longer than those who didn’t participate in the program. Also when quality of life was considered in the estimations, the model showed that IDUs who receive MMT, would live 2.12 QALYs longer than non-recipients. Our estimated life years and QALYs lived are lower than those reported by similar studies. This can be explained, to some extent, with the amount of SMR used in this study. We used the SMR of IDUs in Asian countries, which is higher than those reported for North America, Eastern Europe and Australia (14.47 compared to 6.4, 9.25 and 11.19) (36). Although, most reports claim that MMT can be effective, but there is a significant difference between effectiveness of MMT activities. This difference could be due to differences of modeling of calculating case averted, prevalence of HCV infection among IDUs population, and frequency of high risk behaviors among target groups.

Also it should be emphasized that much of the evidence for harm reduction interventions is based on observational study designs, which recruit IDUs and record whether they have been exposed to a specific risk factor under investigation and relate this information to different outcomes such as HCV and HIV transmission. These aren’t optimal designs to test questions of whether an intervention is effective or not. This is because apparent effects of the intervention are often confounded by factors associated with receipt of the intervention. That is, the characteristics of IDUs exposed and unexposed to the intervention can be very different and this can dilute, exaggerate or reverse the true relationship between intervention and outcome. Moreover although the number of sexual contacts, homo or hetero, were different for recipients and non-recipients of the MMT program, yet most of the QALYs gained were due to reduction in sharing injection. This can be explained by higher risk of transmission of HCV infection in sharing injections compared to unprotected sexual contacts (51-54). The risk of HCV transmission by sexual contact is very low (5*10^-7^) (28, 29). In addition, our previous study showed that the number of high risk sexual contacts in the IDUs was lower than high risk injections (20). Our results in this regard are in accordance with other relevant studies (19, 27) Finally the Tornado diagram revealed that our model outcome was more sensitive to discount rate, HCV transmission rate per injection, number of shared individuals in each party and utility weight of different health states in patients with hepatitis C than other parameters. Precise estimation of data on the size of the IDUs population and HCV prevalence among them could be useful in estimation of the total QALYs saved through implementation of MMT in Iran. Estimating the size of the IDU population is difficult because drug use is an illegal and stigmatized activity. Also HCV prevalence is difficult to estimate because many areas lack the capacity to systematically monitor HCV infections among IDUs. Although institutions such as prisons, jails, and drug abuse treatment centers collect most of the data on HCV prevalence, yet they do not necessarily represent the IDU population at large.

However, using local high-risk behavior data to estimate the number of new cases of HCV among IDUs has made our results more applicable in local health policy making. Nonetheless our model has several limitations that merit consideration in interpreting results. Firstly, some of the model inputs are based upon our data, which have been directly gathered from participants by an interviewer-administered questionnaire. A previous study has demonstrated that IDUs may under-report stigmatized behaviors, such as needle sharing and sexual behavior (55). Secondly, although local data sources were used wherever possible to ensure a high level of internal validity, yet input data for utility and transition probability were derived from international literature. These variables may therefore be different for Iranian patients. Nonetheless efforts were made to determine the sensitivity of our results to both structural and parameter uncertainties. In conclusion MMT has been used for the treatment of addiction to heroin and other opiates for a long time and has proven to be safe even when administered for 15 years or longer (56). Also MMT may be safely continued in the presence of stable chronic liver disease (57, 58). Considering its effectiveness and safety, it is appropriate to continue MMT over years, in patients with chronic HCV infection. IDUs not infected with HCV, who enter a MMT program and do not use other drugs or alcohol, are very likely to remain HCV-negative. Beside, given that many current and former MMT clients share injection material with other IDUs, therefore MMT is effective when broadly applied to a large fraction of active IDUs. Although it could be argued that offering MMT to a wider range of IDUs will be associated with compliance problems. Nevertheless, compliance could be addressed by including educational and motivation strategies in MMT programs.
